# Connected health in US, EU, and China: opportunities to accelerate regulation of connected health technologies to optimize their role in medicines development

**DOI:** 10.3389/fmed.2023.1248912

**Published:** 2023-08-07

**Authors:** Susan Awad, Lina Aljuburi, Rebecca S. Lumsden, Marya Mpandzou, Roelie Marinus

**Affiliations:** Global Regulatory Affairs, Sanofi, Paris, France

**Keywords:** digital health, connected health, decentralized clinical trial, regulatory harmonization, artificial intelligence

## Abstract

Connected health technology is playing an increasingly important role in healthcare. The power, complexity, functionality, and accessibility of connected health technologies are increasing rapidly, showing promise for improved and more equitable healthcare outcomes. They are integral to the lifecycle of medical products, from discovery and development to manufacturing and ultimately to the patient. The spectrum of integration between medical products and digital technologies ranges from non-drug specific solutions for supporting adherence or patient monitoring, which may or may not require regulatory approval, to digital therapeutics and software-containing combination products, which make claims supported by clinical evidence. The exponential increase and rapid evolution of connected health technologies – and the accompanying possibilities for innovative healthcare interventions, delivery systems, and clinical trial designs – pose new and complicated regulatory challenges. Currently, connected health may involve the use of regulated medical devices, including software as a medical device, or consumer products, such as wearables or apps, that fall under regulatory discretion. In this paper we examine how connected health technologies intersect with the development and lifecycle of medical products, how they are impacted by existing regulatory frameworks in the US, EU, and China, and propose future focus areas of activity.

## Introduction

Connected health technology is playing an increasingly important role in healthcare. In this paper, we refer to connected health as the use of information technology, digital networks, artificial intelligence (AI) and machine learning (ML) to collect, share, and analyze data on individuals’ health. Connected health technology is used to help patients, healthcare professionals, and health authorities make better-informed healthcare decisions and improve health outcomes. It differs from digital health in that it describes a system “where devices, services or interventions are designed around the patient’s needs” (1), however digital health can be seen as a key subset of connected health activities.

**Figure 1:**
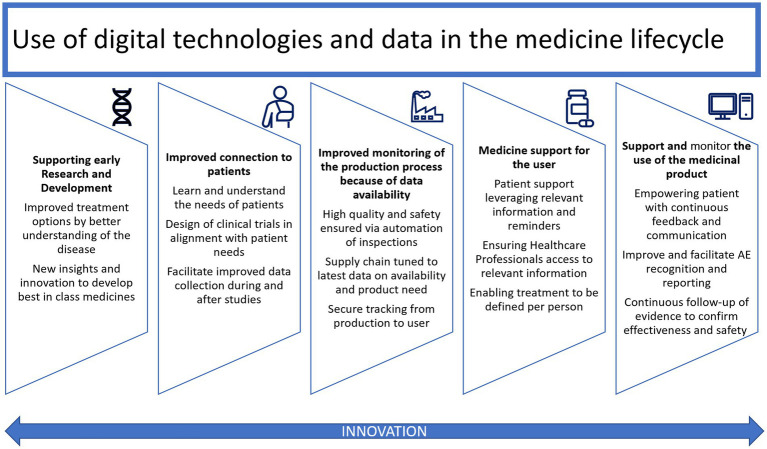
Use of digital technologies and data in the medical product lifecycle, adapted from EFPIA [Ref: Digital Health (efpia.eu) Accessed i28 April 2023].

The power, complexity, functionality, and accessibility of connected health technologies are increasing rapidly, showing promise for improved and more equitable healthcare outcomes. They are integral to the lifecycle of medical products, from discovery and development to manufacturing and ultimately to the patient (See Figure 1). The spectrum of integration between medical products and digital technologies ranges from non-drug specific solutions for supporting adherence or patient monitoring, which may or may not require regulatory approval, to digital therapeutics and software-containing combination products, which make claims supported by clinical evidence. The exponential increase and rapid evolution of connected health technologies – and the accompanying possibilities for innovative healthcare interventions, delivery systems, and clinical trial designs – pose new and complicated regulatory challenges (2). Currently, connected health may involve the use of regulated medical devices, including software as a medical device, or consumer products, such as wearables or apps, that fall under regulatory discretion (3).

In this paper we examine how connected health technologies intersect with the development and lifecycle of medical products, how they are impacted by existing regulatory frameworks in the US, EU, and China, and propose future focus areas of activity.

The use of digital technologies and data is possible and relevant in all stages of the medical product lifecycle: from early phase scientific research where AI and ML may contribute to analyzing large and complex data sets to allowing the use of digital endpoints, decentralized clinical trials to enhanced reporting. These innovations can also democratize research by allowing patients who may not be able to physically or financially access a trial site to participate as well as allowing enhanced reporting for evidence generation from real world data.

## Spotlight on regulatory framework – US

**Figure 2:**
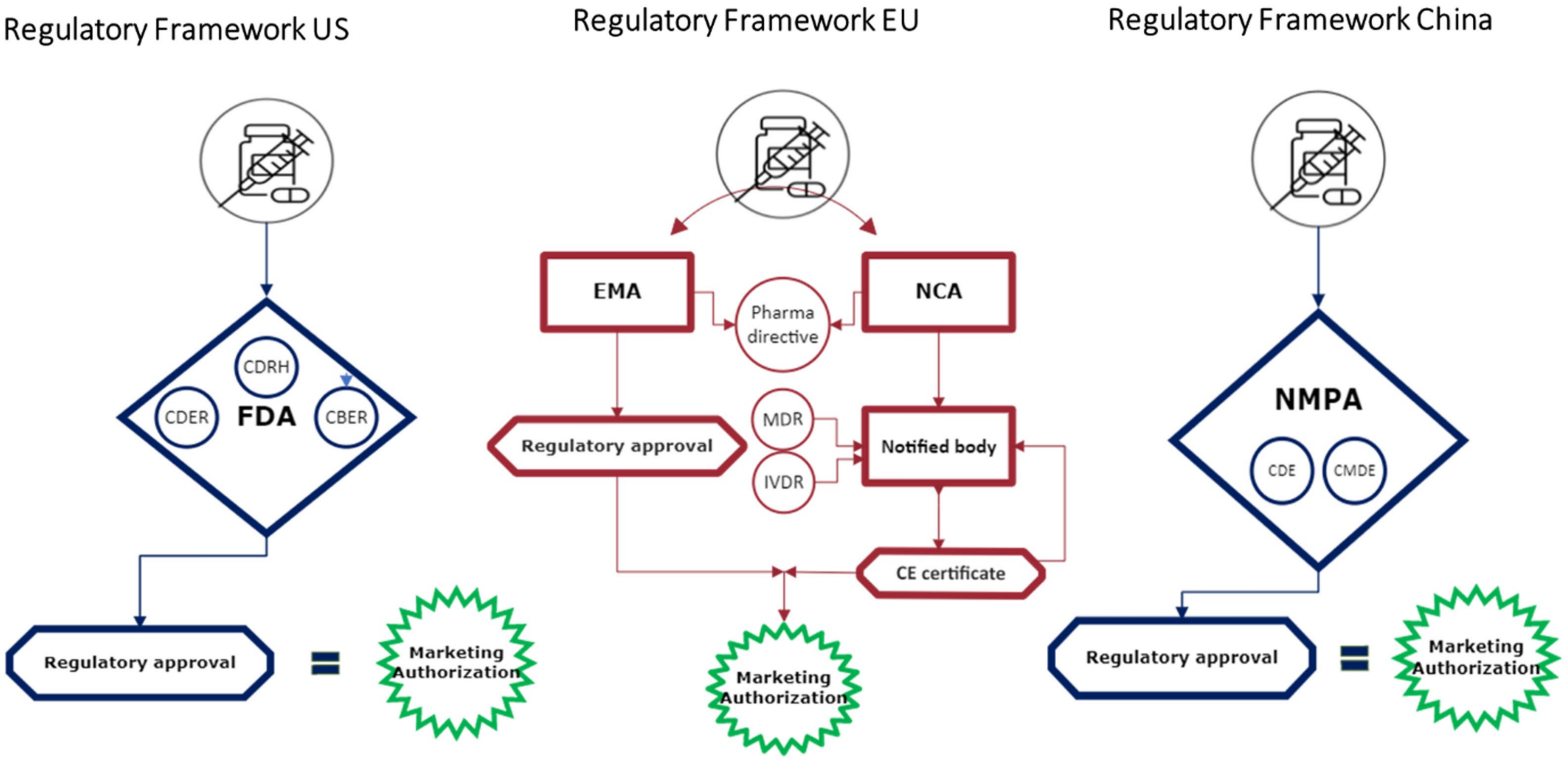
Regulatory frameworks.

Connected health technologies do not fit neatly into existing regulatory pathways (Figure 2). In the U.S., the FDA regulates these technologies in a few ways, depending on their characteristics. Connected health products may be regulated as a diagnostic, a traditional medical device, as “software as a medical device” (SaMD), or as a combination product. Other products may fall under FDA’s regulatory discretion. Recognizing the increasing role that digital health technologies and connected systems are playing in medical product development, in September 2020, FDA launched the *Digital Health Center of Excellence (DHCoE)* within the Center for Devices and Radiological Health (CDRH) to provide centralized expertise and serve as a resource to industry, the public, and FDA staff (4). It has developed a Digital Health Policy Navigator to help product developers understand whether a software function may be subject to regulatory oversight (5).

The definition of *a medical device*, found in Section 201(h) of the Federal Food, Drug, and Cosmetic Act, is very broad. It describes a medical device as “any instrument, machine, contrivance, implant, *in vitro* reagent that’s intended to treat, cure, prevent, mitigate, diagnose disease in man” (6), including everything from a tongue depressor to a robotic surgical device. Devices such as pacemakers and insulin pumps often connect to the internet, hospital networks, the patient’s mobile phone, or other devices to share data, allowing for remote monitoring and real-time treatment adjustments. Because implantable and wearable medical devices are now commonly connected to the internet or other networks, in December 2022 Congress passed legislation requiring sponsors of internet-connected device applications or submissions to provide assurance that the device is cyber-secure and submit a plan to FDA for how it will monitor and address cybersecurity vulnerabilities (7).

Not only is software now commonly embedded in hardware devices, but software itself can be considered a medical device if it meets certain criteria. *Software as a Medical Device (SaMD)* is defined by the International Medical Device Regulators Forum (IMDRF) as “software intended to be used for one or more medical purposes that perform these purposes without being part of a hardware medical device” (8). The IMDRF is a voluntary group of medical device regulators who are working on harmonization of medical device regulation. Within the IMDRF, the FDA chairs the SaMD Working Group, which was formed in 2013 to develop guidance supporting innovation and timely access to safe and effective SaMD (9).

As connected medical devices and SaMD advance and proliferate, health authorities, including the FDA, are exploring how best to ensure their safety, efficacy, and security. In September 2022, FDA completed its *Software Precertification (Pre-Cert) Pilot Program*, which explored innovative approaches to regulatory oversight of medical device software developed by manufacturers who have demonstrated a robust culture of quality and organizational excellence (10). Based on insights gleaned from the pilot, FDA concluded that the rapidly evolving technologies in medical devices could benefit from a new regulatory paradigm, which would require legislative change.

*Combination products* are therapeutic and diagnostic products that combine two or more different regulated components such as drugs, devices, and/or biological products. A drug or biologic used with software may be considered a combination product if the software is considered a medical device. In January 2022, FDA issued final guidance on Principles of Premarket Pathways for Combination Products (11). The guidance outlines three review pathways for combination products based on the mode of action “expected to make the greatest contribution to the overall intended therapeutic effects,” known as the primary mode of action (PMOA). Based on the PMOA, the Agency will assign each combination product to a lead Center that will have primary jurisdiction for premarket review and regulation.

Perhaps the most advanced and complex regulatory questions are raised by medical products that incorporate or rely on *artificial intelligence and machine learning (AI/ML)*. In January 2021, the DHCoE released the Agency’s first AI/ML-Based SaMD Action Plan, which describes FDA’s approach to oversight of AI/ML-based SaMD (12). To advance its thinking about the challenges related to AI/ML in drug development, in May 2023, the Agency released a public discussion paper, “Using Artificial Intelligence and Machine Learning in the Development of Drug and Biological Products” (13), which provides an overview of the current and potential future uses for AI/ML in therapeutic development. It also discusses the possible concerns and risks associated with these innovations and how to address them.

Finally, digital health technologies are increasingly being used as tools to collect data and monitor subjects in clinical trials evaluating other investigational products. This use, in which the digital health technology is not the primary regulatory subject, raises a separate set of regulatory questions. Digital health technologies can enable decentralized or hybrid clinical trials through the remote collection of patient data, as well as deeper insight into a product’s safety and effectiveness through real-time measurements of symptoms or digital biomarkers. FDA issued draft guidance addressing the remote acquisition of data in clinical investigations through digital health technologies (DHT) in December 2021 (14). “The guidance addresses issues including selection of DHTs for remote data collection, verifying and validating their accuracy and reliability, relating their findings to end points, ensuring their usability and acceptability by study participants, and identifying and managing their risks in clinical investigations including privacy risks” (15). In March 2023, FDA published a Framework for the Use of DHTs in Drug and Biological Product Development to guide the use of DHT-derived data in regulatory decision-making for drugs (16).

## Spotlight on regulatory framework – EU

Most connected health solutions and technology are regulated as *devices* and/or *combination products* in the EU. Different and sometimes multiple regulations may apply for one application depending on the use and characteristics. Products or equipment intended for a medical use are regulated as medical devices and must undergo a conformity assessment to demonstrate safety and performance. Drug-device ‘combination products’ do not have a unique category; instead, they are characterized as containing both a drug and a device and face different, parallel regulatory pathways. Whereas drugs in Europe are usually reviewed and approved by the European Medicines Agency (EMA), medical devices are reviewed at member state level by designated notified bodies.^1^ Both pathways have different timelines as well as different regulators. For combination products, if the principal intended purpose is for the medicine, it is seen as a medicinal product that includes a medical device and the entire product is regulated under EU Pharmaceutical legislation (17, 18). In these cases, the EMA is involved in the review process for the medical device; however, it may still require a conformity assessment to ensure compliance.

Since 2021 a new Medical Devices Regulation (MDR) and *In Vitro* Diagnostic Regulation (IVDR) regulation are applicable within the EU, followed by an initial 4-year transition period, repealing previous legislation (19, 20). The MDR changed the European legal framework for certain categories of medical devices and introduced new principal and supportive responsibilities for EMA and for national competent authorities (NCA) in the assessment of certain categories of products. Obtaining a Notified Body Opinion (NBOp) requires a significant investment of time and resources directly impacting the costs and time to approval.

For certain high-risk medical devices, notified bodies are requested to consult expert panels before they can issue a CE (“conformité européenne”) certificate (21). Expert panels have been designated in the Commission Implementing Decision (EU) 2019/1396 and consist of high-level experts appointed because of their level of expertise following a call for expression of interests (22). *The new IVDR* replaces the 1998 *In Vitro* Diagnostic Directive (IVDD) (20, 23) and is more complicated and comprehensive than the IVDD. A major difference is the change from Directive to Regulation which removes room for local interpretation on implementation at a national level. The European Commission (EC) has acknowledged possible disruptions in the supply of both IVD’s and medical devices due to limitations in notified bodies capacity and delays caused by the pandemic, leading to a recent extension of the transition period for full implementation (24).

Software can also fall under the two regulations if it is “Software that is intended to be used, alone or in combination, for a medical purpose as specified by the European Commission’s Medical Device Coordination Group (MDCG) (25). The definition and criteria for *software qualification* was published and endorsed in a guidance in 2019, the MDCG 2019–11 (26). This document describes qualification criteria, which software is considered a medical device, and its risk classification. The MDCG guideline is not legally binding but presents a common understanding aiming at an effective and harmonized implementation of the legislation.

However, software for a given set of human-defined objectives, generating outputs such as content, predictions, recommendations, or decisions influencing the environments they interact with could be considered Artificial Intelligence (AI) (27).

In April 2021 the EC proposed a global first legal framework for AI (28) to address both the challenges and opportunities of this new technology. This new regulation, the Artificial Intelligence Act (AIA), is a risk-based approach legislation to guarantee the fundamental rights of people and businesses but also aims to be future proof as AI is a vast evolving technology (29). The four levels of risks described in the AI consist of unacceptable, high, limited and minimal/no risk. This proposal comes together with a coordinated plan which represents a joint commitment between the EC and Member States to maximize AI potential and enable Europe to compete globally. After a transition period the regulation will become applicable at the earliest in 2024. However numerous discussions have taken place on the AIA, where the agreed definition of AI is “a system developed through machine learning approached and logic-and knowledge-based approaches” which ensures a clear distinction between a classical software system and AI (30). Flexibilities have also been built in to add new techniques and specificities to ensure the AI act remains future proof.

In addition to the AI act, an AI liability Directive was adopted in 2022 following a recommendation in the white paper “White Paper on Artificial Intelligence: a European approach to excellence and trust” (31, 32).

As connected health and digitalization within the healthcare landscape accelerates, the EC has proposed a European Health specific ecosystem with common standards, practices, rules infrastructure and a governance framework, the European Health Data Space (EHDS) (33). The objective of the EDHS is not only to empower people through increased digital access but also to empower people to get better control of their personal health data and foster a single market for electronic health record systems, medical devices and high-risk artificial intelligence systems, while ensuring a framework, for the use of health data in research, innovation, policy making and regulatory activities (34). The European Medicines Regulatory Network are engaging through the work of the HMA/EMA joint Big Data Steering Group (35).

Finally, the EC, the Heads of Medicines Agencies (HMA) and the EMA published a ‘Recommendation paper on decentralized elements in clinical trials’ in December 2022. The paper seeks to “address the roles and responsibilities of the sponsor and investigator, electronic informed consent, IMP delivery, trial related procedures at home, data management and monitoring in a decentralized clinical trial setting” while also providing “an overview of the current national provisions applicable in each Member State (MS) in relation to these topics…” (36). The EMA has also published a Questions and answers: Qualification of digital technology-based methodologies to support approval of medicinal products document in 2020 (37).

## Spotlight on regulatory framework – China

Like other regions, connected health solutions are currently regulated as a medical device or a combination product in China. The Chinese Center for the Evaluation of Medical Devices (CMDE) is in charge of the technical review of the medical device and the National Medical Products Administration (NMPA) provides the final approval and authorization (38, 39).

For combination products, the CDE and CMDE will conduct a joint review. First the applicant gets a classification confirmation, whether the drug or the device is seen as the key therapeutic function. When the combination product is identified as a product where the drug provides the therapeutic effect, the CDE will lead the overall technical review, and vice –versa in cases when the device part is identified as the lead element. After the review the NMPA will undertake the final authorization.

Digital therapy is still at an early stage and listed as new technology in the NMPA Regulatory Science Plan 2021, to meet industry development needs (40). Though no definitions or specific regulations are available yet, the initial discussions have started to formulate a regulatory framework.

For AI there is also not yet a formal definition or specific regulation. However, both Health Authority and senior government departments are actively looking into this topic (41, 42). In the absence of dedicated guidance, the medical device guidance can be used to register the AI used as software.

## Discussion

The regulatory frameworks for connected health technologies are beginning to mature, but they are doing so within the contexts of disparate regional systems. Each major health authority is taking a slightly different approach to regulating connected health technologies, largely based on legacy approaches to traditional medical device regulation. These new technologies are revolutionizing both drug development and healthcare delivery, and their effective regulation will require new frameworks and globally aligned thinking. Regulators are already undertaking this important work together through fora such as the International Coalition of Medicines Regulatory Authorities (ICMRA), which has issued recommendations to help regulators to address the challenges that the use of AI poses for global medicines regulation (43).

To realize the promise of connected health solutions for improving patient outcomes and health equity globally, regulators, advocates, and industry sponsors will need to work together to streamline, clarify, and reinforce the regulatory pathway for these new tools. Three critical areas should be progressed: (1) optimize the process for validating new technology and tools, (2) upskill and expand the regulatory workforce to close gaps in expertise, and (3) increase knowledge-sharing among regulators to provide a foundation for future convergence and harmonization.

1. Validating new digital technologies, whether they are tools for remote data collection in clinical trials or algorithms used in clinical care, is complex, often requiring additional test data and periodic testing. Learning algorithms that evolve over time and cybersecurity advances may necessitate ongoing review and repeated validation exercises, which may not align with regulators’ current workflow, capacity, or authority. One approach may be to establish and equip offices of AI Assurance within health authorities to test and re-validate software over the total product lifecycle. Similarly, a new health AI coalition in the US, which includes FDA as an observing member, has called for the creation of a registry of health AI tools and an independent assurance accreditation lab to evaluate them (44).
2. Health authorities will also need new expertise as they are asked to review and regulate ever more sophisticated connected health technologies. Currently, in the EU, notified bodies may be requested to consult an expert panel, and in the US, the FDA may convene an Advisory Committee to provide outside expertise. But these consultative processes are time-and resource-intensive and are not conducive to a total product lifecycle approach to review. Health authorities will need the resources to train and hire professionals with the requisite skill sets to provide in-house, ongoing advice regarding connected health safety, quality, and effectiveness. This emphasizes the need and opportunity to join forces and intensify collaboration between health authorities and industry to ensure the right skills and expertise are being developed.
3. By their very nature, connected health technologies present global regulatory questions. To stay abreast of a rapidly evolving landscape and ensure consistency in regulatory approaches to new technologies, health authorities should increase efforts toward knowledge-sharing and alignment on principles and terminology in this space. FDA’s leadership role in the IMDRF SaMD working group and the EMA’s international work through the Big Data workplan are a promising start, but this work needs to be elevated and expanded to include other connected health technologies that are rapidly changing the practice of medicine.

The breakneck pace of innovation in the connected health space is thrilling and holds enormous potential for better, more equitable health outcomes for patients across the globe. We need to ensure our regulatory structures, systems, and stakeholders are prepared to facilitate adoption and assure the quality of these technologies to maximize their impact and realize their promise.

## Author contributions

SA and RM conducted research and drafted the article’s content. RL and LA reviewed and edited drafts, provided strategic direction, and oversight for the manuscript. MM conducted research and assisted with references. All authors contributed to the article and approved the submitted version.

## Conflict of interest

The authors declare that the research was conducted in the absence of any commercial or financial relationships that could be construed as a potential conflict of interest.

## Publisher’s note

All claims expressed in this article are solely those of the authors and do not necessarily represent those of their affiliated organizations, or those of the publisher, the editors and the reviewers. Any product that may be evaluated in this article, or claim that may be made by its manufacturer, is not guaranteed or endorsed by the publisher.

## Footnotes

^1^ Notified Bodies (europa.eu)
